# Supplementary material for: Hot spot prediction in protein-protein interactions by an ensemble system
Source: BMC Syst Biol. 2018 Dec 31;12(Suppl 9):132. doi: 10.1186/s12918-018-0665-8 (PMC6311905; doi:10.1186/s12918-018-0665-8)
Supplement: Supplementary file 2 — All relevant MATLAB code. A simple Matlab implement of our predictor. (ZIP 19,742 kb) [file 12918_2018_665_MOESM2_ESM.zip › Additional file 2/Read ASA/ASA website/dbMPIKT/1KBHB.html]

 NetSurfP - Protein Surface Accessibility and Secondary Structure Predictions

  

|  |  |  |
| --- | --- | --- |
|  |  | NetSurfP - Protein Surface Accessibility and Secondary Structure Predictions  Technical University of Denmark |

  


---

```
# For publication of results, please cite:
# A generic method for assignment of reliability scores applied to solvent accessibility predictions.
# Bent Petersen, Thomas Nordahl Petersen, Pernille Andersen, Morten Nielsen and Claus Lundegaard
# BMC Structural Biology 2009, 9:51 doi:10.1186/1472-6807-9-51
#
# Column 1: Class assignment - B for buried or E for Exposed - Threshold: 25% exposure, but not based on RSA
# Column 2: Amino acid
# Column 3: Sequence name
# Column 4: Amino acid number
# Column 5: Relative Surface Accessibility - RSA
# Column 6: Absolute Surface Accessibility
# Column 7: Z-fit score for RSA prediction
# Column 8: Probability for Alpha-Helix
# Column 9: Probability for Beta-strand
# Column 10: Probability for Coil
E P  1KBHB                  1    0.787 111.619  -0.419   0.003   0.003   0.994
E N  1KBHB                  2    0.655  95.863  -2.470   0.016   0.005   0.979
E R  1KBHB                  3    0.683 156.315  -1.903   0.053   0.005   0.942
E S  1KBHB                  4    0.595  69.699  -1.176   0.109   0.005   0.886
B I  1KBHB                  5    0.264  48.840  -0.730   0.321   0.003   0.675
E S  1KBHB                  6    0.258  30.226  -0.145   0.246   0.004   0.750
E P  1KBHB                  7    0.540  76.697   0.387   0.858   0.002   0.139
E S  1KBHB                  8    0.528  61.940   0.058   0.923   0.002   0.076
B A  1KBHB                  9    0.114  12.563   0.021   0.970   0.001   0.030
B L  1KBHB                 10    0.048   8.770   0.190   0.970   0.001   0.030
E Q  1KBHB                 11    0.367  65.600   1.136   0.970   0.001   0.030
E D  1KBHB                 12    0.482  69.499   1.285   0.970   0.001   0.030
B L  1KBHB                 13    0.044   8.038   0.401   0.970   0.001   0.030
B L  1KBHB                 14    0.108  19.738   0.244   0.970   0.001   0.030
E R  1KBHB                 15    0.497 113.882   0.864   0.970   0.001   0.030
B T  1KBHB                 16    0.228  31.610  -0.063   0.923   0.002   0.076
B L  1KBHB                 17    0.078  14.227  -0.052   0.694   0.003   0.303
E K  1KBHB                 18    0.522 107.458  -0.408   0.321   0.003   0.675
E S  1KBHB                 19    0.388  45.474  -0.307   0.053   0.005   0.942
E P  1KBHB                 20    0.414  58.704  -1.538   0.016   0.005   0.979
E S  1KBHB                 21    0.632  74.117  -2.127   0.016   0.005   0.979
E S  1KBHB                 22    0.367  43.059  -0.101   0.016   0.005   0.979
E P  1KBHB                 23    0.462  65.501  -0.993   0.694   0.003   0.303
E Q  1KBHB                 24    0.601 107.374  -0.417   0.858   0.002   0.139
E Q  1KBHB                 25    0.365  65.189  -0.008   0.923   0.002   0.076
B Q  1KBHB                 26    0.203  36.292  -0.511   0.923   0.002   0.076
E Q  1KBHB                 27    0.491  87.746  -0.092   0.923   0.002   0.076
E Q  1KBHB                 28    0.408  72.833   0.588   0.938   0.007   0.055
B V  1KBHB                 29    0.056   8.546   0.489   0.938   0.007   0.055
B L  1KBHB                 30    0.115  21.038   0.432   0.975   0.003   0.022
E N  1KBHB                 31    0.444  65.016   0.577   0.938   0.007   0.055
B I  1KBHB                 32    0.086  15.836   0.450   0.938   0.007   0.055
B L  1KBHB                 33    0.093  16.955  -0.206   0.802   0.014   0.185
E K  1KBHB                 34    0.501 103.076   0.013   0.522   0.016   0.462
E S  1KBHB                 35    0.372  43.540  -1.080   0.246   0.004   0.750
E N  1KBHB                 36    0.310  45.428  -0.307   0.053   0.005   0.942
E P  1KBHB                 37    0.332  47.097  -0.723   0.782   0.003   0.216
E Q  1KBHB                 38    0.490  87.460   0.373   0.923   0.002   0.076
B L  1KBHB                 39    0.089  16.314   0.112   0.970   0.001   0.030
B M  1KBHB                 40    0.056  11.206   0.638   0.970   0.001   0.030
B A  1KBHB                 41    0.168  18.547  -0.863   0.988   0.000   0.012
B A  1KBHB                 42    0.198  21.787  -0.387   0.970   0.001   0.030
B F  1KBHB                 43    0.073  14.691  -0.069   0.970   0.001   0.030
B I  1KBHB                 44    0.058  10.674   0.565   0.970   0.001   0.030
E K  1KBHB                 45    0.359  73.785   1.069   0.970   0.001   0.030
E Q  1KBHB                 46    0.416  74.298   0.318   0.923   0.002   0.076
B R  1KBHB                 47    0.209  47.838  -0.082   0.858   0.002   0.139
B T  1KBHB                 48    0.115  15.923  -0.746   0.782   0.003   0.216
E A  1KBHB                 49    0.389  42.846  -0.668   0.802   0.014   0.185
E K  1KBHB                 50    0.533 109.556   0.634   0.802   0.014   0.185
B Y  1KBHB                 51    0.179  38.252  -0.081   0.717   0.014   0.269
E V  1KBHB                 52    0.279  42.867   0.026   0.622   0.015   0.363
E A  1KBHB                 53    0.598  65.889  -0.761   0.430   0.016   0.555
E N  1KBHB                 54    0.658  96.302  -1.054   0.246   0.004   0.750
E Q  1KBHB                 55    0.498  88.979  -0.408   0.053   0.005   0.942
E P  1KBHB                 56    0.530  75.136  -1.484   0.016   0.005   0.979
E G  1KBHB                 57    0.626  49.227  -2.094   0.018   0.019   0.964
E M  1KBHB                 58    0.433  86.663  -1.115   0.018   0.019   0.964
E Q  1KBHB                 59    0.865 154.525  -0.055   0.003   0.003   0.994

Explain the output.  Go back.
```

---
